# Great Ormond Street Children's Hospital

**Published:** 1892-06-04

**Authors:** 


### HOSPITAL ADMINISTRATION.

### GREAT ORMOND STREET CHILDREN'S HOSPITAL.

Important Alterations in the Rules.

At a special Court of the Governors of the Great Ormond Street Hospital, held after the annual general meeting on the 31st ult., certain important alterations in the rules affecting Governors' letters of recommendation were under consideration. The Duke of Fife, the President of the hospital, took the chair.

Mr. Arthur Lucas, chairman of the Committee of Management, in moving the resolutions given below, briefly stated the reasons which had led the Committee to take action in the matter. The system followed by the hospital had been the dual system of in-patient and out-patient letters of recommendation prevalent at most other hospitals. The disadvantages of such a system had been found to be great, for, as a matter of fact, the hospital, in common with most other hospitals, admitted, immediately it was brought, any case which was suffering from illness. If only a slight case, it was treated as an out-patient, and if serious and there was room, it was at once admitted as an in-patient. It was, therefore, found in practice that children for the most part were admitted without letters at all, and as that gave rise to complaint on the part of certain of the subscribers and Governors, the Committee, after very careful consideration and after consultation with the Governors, had decided to recommend the doing away with the dual system of letters altogether. In its place one letter only (the form of which was still under consideration, but which would be very simple) would be issued for both in and out patients. The patients would be received to the hospital on any day between certain hours, be seen at once by the medical officers, and in their opinion a case for admission, be at once taken into the wards, provided there be room. If not thought serious enough for admission as an in-patient, the case would be placed on the out-patient list. The Committee claimed for the plan simplicity of working, and the allaying of any slight friction in the working of the hospital which might at present exist.

Mr. John Murray seconded, and the resolutions were agreed to.

The resolutions were as follows:—

(1) That in future, there shall be only one form of letter recommending children for treatment at the hospital, and that cases so recommended shall, if suitable, be admitted at the discretion of the medical officers. (2) That special provision be made to facilitate the admission, as in-patients, of country cases (namely, those resident beyond a radius of ten miles from the hospital). [This provision was made by the alteration of paragraph 3, rule 194, as follows: "To prevent disappointment, a subscriber desirous of obtaining admission for a child from the country is requested, before sending the child to the hospital, to apply to the House Physician for a form of medical certificate, and, having had this certificate filled up and signed by a medical man who has seen the child, to return it to the House Physician, in order that the medical officers may be able to judge whether the case is a suitable one for admission. The House Physician will at once inform the subscriber whether the case is eligible for admission, and, if so, how soon it can be admitted."] (3) That children who are brought with subscribers' letters shall have precedence, except in cases of special urgency. (4) That, in future, the number of subscribers' letters issued annually by the Secretary shall be according to the following scale: Donors of £100 or upwards, and every subscriber of £10 10s., or more annually 12 letters, and additional letters of recommendation on application at any time; donors of £50 or upwards, and every subscriber of £5 5s. and upwards annually, 12 letters; life governors (donors of £31 10s.) and subscribers of £3 3s., six letters; life donors (donors of £21) and subscribers of £2 2s., three letters; life donors (donors of £10 10s.) and subscribers of £1 1s., two letters.

The necessary alterations in the rules to enable the foregoing scheme to be carried out were agreed to.

---

